# From awareness to action: exploring health information seeking behavior in coronary heart disease patients: a cross-sectional study

**DOI:** 10.3389/fpubh.2026.1749036

**Published:** 2026-01-27

**Authors:** Yunyu Guo, Panpan Tang, Meijuan Lan, Yuping Zhang, Xueqing Wang, Yueying Jiang, Yue Zhao, Maidiniguli Maitikuerban, Manjun Wang, Jing Shao, Dandan Chen, Zhihong Ye, Leiwen Tang

**Affiliations:** 1 Department of Nursing, The Second Affiliated Hospital of Zhejiang University School of Medicine, Hangzhou, Zhejiang, China; 2 Department of Endocrinology, Peking University Shenzhen Hospital, Shenzhen, Guangdong, China; 3 Sir Run Run Shaw Hospital, Zhejiang University School of Medicine, Hangzhou, Zhejiang, China; 4 School of Nursing and Institute of Nursing Research, School of Medicine, Zhejiang University, Hangzhou, Zhejiang, China

**Keywords:** coronary heart disease, health information seeking, risk perception, risk perception attitude framework, self-efficacy

## Abstract

**Introduction:**

Coronary heart disease (CHD) is a major global health burden requiring long-term management. Despite the essential role of health information seeking behavior (HISB) in disease self-management, current levels among CHD patients remain low, and research on its influencing factors is limited.

**Objectives:**

This study aimed to explore HISB among patients with CHD and to identify factors associated with variations in HISB using the Risk Perception Attitude (RPA) framework.

**Methods:**

A cross-sectional study of 330 CHD patients was conducted in China, using convenience sampling method. Data were collected through validated questionnaires assessing sociodemographic and clinical characteristics, HISB, risk perception, and self-efficacy. K-means clustering based on the RPA framework was employed to empirically identify distinct patient subgroups. Multivariate linear regression identified factors associated with of HISB within each subgroup.

**Results:**

Four distinct subgroups were identified based on risk perception and self-efficacy: Responsive (6.7%), Proactive (41.2%), Indifference (8.2%), and Avoidance (43.9%). Multivariate regression revealed subgroup-specific factors: for Responsive, physical diagnosis and treatment risk was significant [β = 2.049, 95%CI (0.528,3.570)]; For Proactive, higher education [β = 4.725, 95%CI (2.272,7.178)], per capita monthly household income and self-efficacy were positively associated, while type of medical insurance [β = −5.814, 95%CI (−8.800, −2.828)], number of other diseases, and economic risk were negative predictors; For Indifference, only type of medical insurance was significant [β = −6.447, 95%CI (−12.503, −0.391)]; For Avoidance, older age was linked to lower HISB [β = −4.757, 95%CI (−8.525, −0.989)], whereas higher education increased it [β = 5.432, 95%CI (2.353, 8.511)].

**Conclusions:**

This study validates the heterogeneity of CHD patients through RPA-based subgrouping, revealing that health information seeking behaviors are driven by distinct psychological and socioeconomic mechanisms across different groups. These findings underscore the limitation of uniform health education approaches and highlight the necessity of implementing subgroup-tailored strategies. By aligning clinical and public health interventions with the specific psychographic profiles of patient groups, healthcare providers can significantly enhance the precision and effectiveness of chronic disease management.

**Registration:**

www.chictr.org.cn, identifier: ChiCTR2300069238.

## Introduction

Coronary heart disease (CHD) is a major global disease burden, affecting millions worldwide (1). As a chronic condition requiring long-term management, effective health information seeking behavior (HISB) is essential for patients to understand their illness and engage in self-care (2). HISB refers to the process by which individuals search for, access, and use information related to their health condition, risk prevention, and disease management. For CHD patients, HISB is instrumental in fulfilling knowledge gaps, mitigating health risks, and enhancing disease management (3), thereby improving quality of life during long-term coexistence of the disease. Nevertheless, despite high information needs (4), many CHD patients exhibit a low level of HISB, suggesting a disconnect between need and action (5).

Existing studies have reported sociodemographic factors such as age, education, gender, health status, and income as predictors of HISB across diverse chronic diseases (6). Yet, evidence specific to CHD patients remains limited, particularly regarding psychological factors that may uniquely influence HISB in this population. Risk perception, a major determinant of individual behavior (7), has been shown to play a significant predictive role in cardiovascular disease self-management (8) and shares a causal relationship with HISB (7, 9). It refers to the attitudes, judgments, and empirical understandings that individuals develop because of irrational, subjective analysis of risk information about their circumstances (8, 10). Over the long term, CHD patients face several non-negligible health risks, and variations in risk perception can lead to different HISB patterns (7). Self-efficacy, the belief in one's ability to effectively manage or prevent health risks, also affects how patients respond to these risks (11). Study shows that the relationship between risk perception and HISB must be examined in the context of people's efficacy beliefs (12).

However, current research lacks the integration and guidance of a systematic theoretical framework, resulting in intervention strategies remaining fragmented and difficult to implement precisely (13). Moreover, there are significant internal differences among patients with coronary heart disease: different individuals exhibit considerable heterogeneity in terms of disease cognition, psychological response, and resource accessibility. If such personalized demands are ignored, it is likely to lead to inefficient “one-size-fits-all” interventions and a waste of resources. The Risk Perception Attitude (RPA) framework provides a new perspective for solving the above problems. This framework takes risk perception and self-efficacy as the core dimensions and classifies the population into four categories: Responsive (high risk perception, high self-efficacy), Proactive (low risk perception, high self-efficacy), Avoidance (low risk perception, low self-efficacy), and Indifferent (high risk perception, low self-efficacy) (14). Compared with other theoretical models, the unique advantage of the RPA framework lies in its ability to comprehensively assess an individual's risk perception level and self-efficacy status, and fully reveal the group differences in healthy behaviors. It not only applies to the psychological and behavioral characteristics of patients with coronary heart disease, but also provides solid theoretical support for formulating precise and differentiated health information intervention strategies.

Despite the growing body of literature on HISB, distinct gaps remain in the context of coronary heart disease. First, previous research among CHD populations has predominantly focused on descriptive analyses of overall HISB levels or employed “variable-centered” approaches to identify isolated sociodemographic predictors. While these studies provide valuable insights into who engages in information seeking—such as establishing associations with age, education, and income (5, 6)—they often overlook the complex psychological interplay between risk perception and efficacy beliefs that explains why these behaviors occur. Second, regarding theoretical application, although the RPA framework has been successfully used to predict HISB in general contexts (15), current research on CHD management often lacks the integration of such systematic frameworks, resulting in intervention strategies that remain fragmented (13).

To bridge these gaps, this study adopts a “person-centered” clustering approach. While similar data-driven segmentation methods (e.g., latent profile analysis) have been effectively explored in patients with type 2 diabetes (2), such approaches remain underexplored within the CHD population. By utilizing K-means clustering based on the RPA framework, this study aims to empirically identify distinct patient subgroups (Responsive, Proactive, Indifference, and Avoidance) and investigate the unique factors associated with HISB within each specific group. This provides a theoretical and empirical basis for developing precise, subgroup-tailored intervention strategies.

## Methods

### Study design

This cross-sectional study aimed to explore HISB among CHD patients and identify factors associated with variations in HISB using the RPA framework. The study was conducted from May to September 2023 in the Department of Cardiology at a tertiary-level hospital in Zhejiang Province, China.

### Participants

Participants were CHD patients aged ≥18 years, with a stable medical condition and clear consciousness, who could provide voluntary participation and informed consent. Patients with severe cardiopulmonary dysfunction, neurological or psychiatric disorders, intellectual disability, or sensory impairments were excluded. A power analysis determined the required sample size. Assuming a medium effect size (Cohen's *d* = 0.5), α = 0.05, and power = 0.8, the sample size was set at 300 participants, with an additional 10% to account for potential dropouts.

### Measures

#### Clinical and sociodemographic characteristics

Data were collected via a self-designed paper questionnaire covering socio-demographic information and disease-related information, such as sex, age, marital status, educational level, occupation, per capita monthly household income, place of residence, type of medical insurance, number of other diseases, and so on.

#### Health information seeking behavior

A specialized instrument, the Chronic Disease Health Information Seeking Behavior Questionnaire, was developed for this study. The rationale for its development was to comprehensively capture the behavioral process, spanning from information needs to evaluation, and to incorporate “passive acquisition.” This represents a critical dimension often overlooked by existing tools that focus solely on active seeking. The questionnaire comprises 30 items across four dimensions. The total HISB score is calculated by summing the scores of three dimensions: Health Information Needs (Items 1–13), Health Information Seeking Abilities (Items 18–23), and Health Information Evaluation (Items 24–30), yielding a total of 26 items with a theoretical range of 26 to 130. The “Seeking Channels” dimension (Items 14–17) is excluded from the total score calculation. This exclusion is based on the rationale that “Channels” represent the mode or source of information access (a descriptive categorical indicator) rather than the latent trait of information-seeking capability or engagement level. Higher total scores indicate a higher level of active engagement in health information seeking. Psychometric testing demonstrated robust validity and reliability. Before the formal survey, a rigorous development process was conducted. First, an expert panel consisting of 10 specialists (including experts in Cardiology, General Practice, Chronic Disease Management, and Nursing Informatics) was convened for two rounds of Delphi consultation. Items were screened based on statistical criteria: those with an expert importance mean score < 4.0 or a coefficient of variation >25% were considered for deletion. Following the expert review, a pilot study was conducted with 30 chronic disease inpatients to assess the clarity and feasibility of the items, confirming that the questionnaire could be completed within 20 min. The scale-level content validity index (S-CVI/UA) was 0.83, with item-level indices (I-CVI) ranging from 0.89 to 1.00. Construct validity was established via Exploratory Factor Analysis (EFA), which yielded a Kaiser-Meyer-Olkin (KMO) value of 0.930 (*P* < 0.001) and extracted three principal components explaining 75.181% of the total variance, with factor loadings between 0.572 and 0.863. The questionnaire exhibited high internal consistency, with an overall Cronbach's α of 0.970 (Supplementary File 1).


$$\begin{array}{r} \text{Total Score}=\overset{13}{\underset{i=1}{\sum }}\left({\text{Needs}}_{i}\right)+\overset{23}{\underset{j=18}{\sum }}\left({\text{Abilities}}_{j}\right)\\ +\overset{30}{\underset{k=24}{\sum }}\left({\text{Evaluation}}_{k}\right) \end{array}$$ (1)

#### Risk perception

The risk perception questionnaire developed by Lei et al. (16) was used to measure the risk perception of chronic disease patients during hospitalization, with a Cronbach's alpha coefficient of 0.884. The questionnaire contains 3 dimensions and 12 entries, namely economic risk (Items 1–4), physical diagnosis and treatment risk (5–9), and psychosocial risk (Items 10–12). A 5-point Likert scale was used, with scores ranging from 12 to 60. The Cronbach's α value in this study was 0.944 (Supplementary File 2).

#### Self-efficacy

The heart health self-efficacy scale, originally developed by Mares et al. and translated into Chinese by Xuemei et al. (17), was used for assessment. It includes two dimensions and 24 items, each rated on a 1–5 scale, with total scores ranging from 12 to 60. The self-efficacy subscale demonstrated good reliability with a Cronbach's alpha of 0.773 in previous studies and showed high internal consistency in this study (Cronbach's α = 0.903) (Supplementary File 3).

### Statistical analysis

The statistical analysis commenced with an exploration of the data's distribution. Descriptive statistics were utilized to encapsulate the characteristics of the overall study population, encompassing sociodemographic, and clinical facets. Variables underwent normality testing; those adhering to a normal distribution were expressed as means and standard deviations, while skewed variables were detailed using medians and interquartile ranges. Categorical variables were represented by frequencies and percentages. Prior to clustering, the total scores of Risk Perception and Self-Efficacy were converted into Z-scores. This standardization ensured that both variables contributed equally to the distance calculation, preventing scale differences from distorting the cluster formation. The rationale for selecting *k* = 4 was supported by both statistical metrics and theoretical relevance (Supplementary Figure 1). Specifically, the Elbow Method indicated a diminishing rate of decline in the Within-Cluster Sum of Squares (Inertia) after *k* = 4, and the Silhouette Analysis showed a local peak at *k* = 4 with an average silhouette coefficient of 0.503. The K-means clustering, executed 25 times within R statistical software (version 4.3.2) to bolster result robustness, bifurcated the dataset into four clusters. Post-clustering, the resultant labels were integrated into the original dataset as a new categorical variable. The ggplot2 package was harnessed to graphically illustrate the clustering outcomes through four quadrants, delineated by the mean values of self-efficacy and risk perception.

Subsequently, each of the four CHD patient subgroups was subjected to descriptive statistical analysis using SPSS 27.0, unveiling insights into subgroup-specific sociodemographic and clinical traits. Normality tests once again dictated the presentation format for continuous variables within each subgroup: normal distributions were captured as means ± standard deviations, and skewed distributions as medians and interquartile ranges. Categorical variables were re-expressed as frequencies and percentages. For univariate analysis, the study employed independent *t*-tests for normally distributed variables when comparing two groups, and Mann-Whitney U-tests for non-normally distributed variables. Multiple groups were compared using one-way ANOVA for normal distributions and the Kruskal-Wallis H-test for non-normal distributions. Significant omnibus tests were followed by *post-hoc* pairwise comparisons using Tukey HSD and the Bonferroni correction to identify specific subgroup differences. Correlation analysis was performed using Pearson's method for normal variables and Spearman's for non-normal variables. Given the skewed distribution of certain continuous variables and to facilitate clinical interpretation for specific demographic stratifications, these variables were converted into categorical variables for the multivariate regression analysis. Rigorous verification of regression assumptions confirmed the model's validity, with continuous variables checked for multicollinearity and categorical variables dummy-coded. This meticulous approach ensured the reliability of regression results, bolstering the understanding of factors influencing HISB across different patient clusters.

## Results

### Sample characteristics

**Figure 1:**
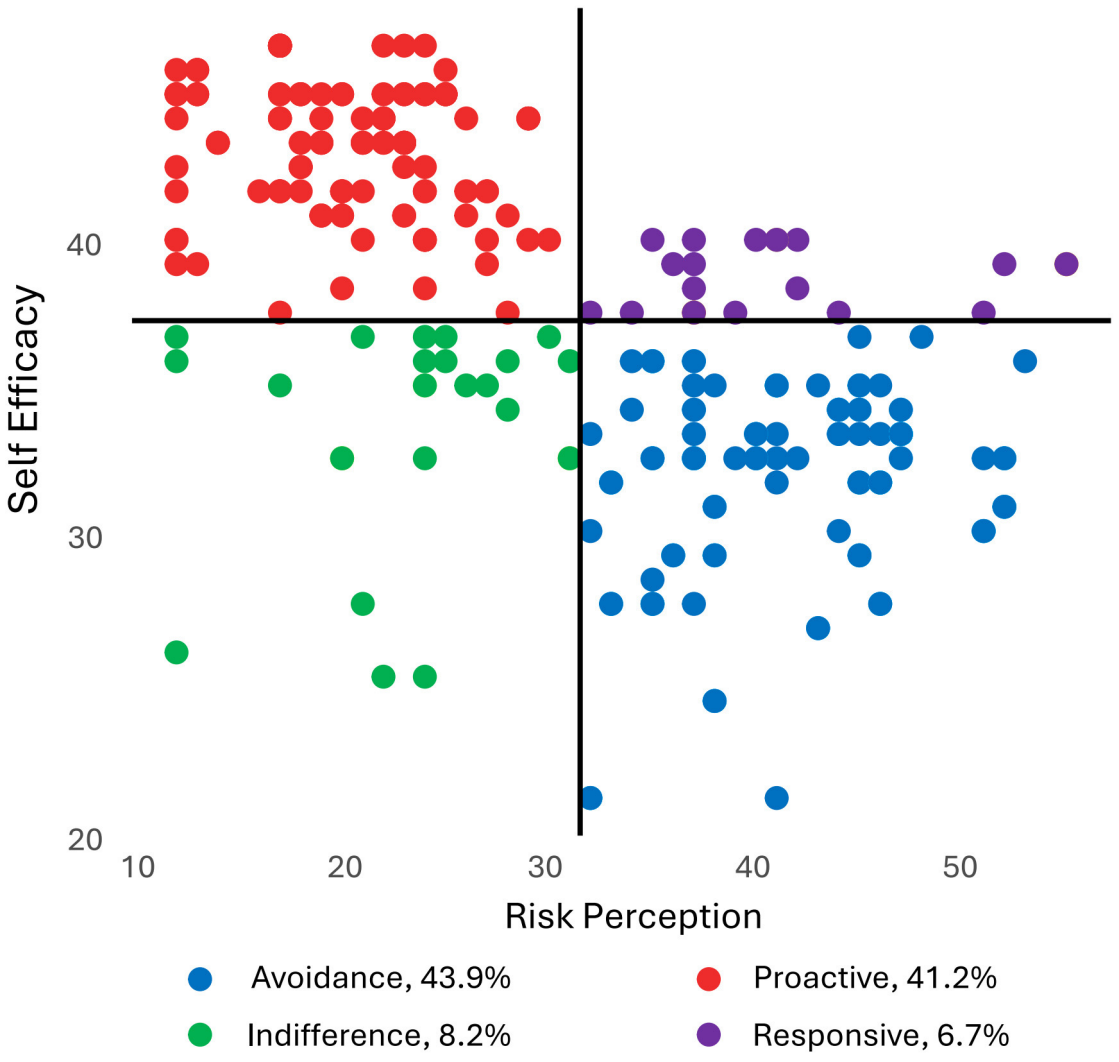
Distribution of four CHD patient subgroups based on the RPA framework. The scatter plot illustrates the classification of participants into four distinct clusters—Responsive (high risk, high efficacy), Proactive (low risk, high efficacy), Indifference (low risk, low efficacy), and Avoidance (high risk, low efficacy)—determined by their standardized scores on Risk Perception (x-axis) and Self-Efficacy (y-axis). The black vertical and horizontal lines represent the cluster centers used to delineate the subgroups.

In this study, a total of 330 CHD patients were recruited, achieving a response rate of 91.7% (330 out of 360). Among the participants, 68.5% were male, with ages ranging from 32 to 89 years. Specifically, 54.8% of the patients were aged between 50 and 69 years, while 36.1% were 70 years or older. They had higher proportions of married, diagnosed ≤ 1 year, experienced PCI surgery, and took regular medication. Based on the RPA framework and the boundaries of the four quadrant divisions obtained from cluster analysis (Self Efficacy mean score = 36.66969, Risk Perception mean score = 31.50303), patients were categorized into four groups, Responsive (*n* = 22, high self-efficacy and risk perception), Proactive (*n* = 136, high self-efficacy and low risk perception), Indifference (*n* = 27, low self-efficacy and risk perception), and Avoidance (*n* = 145, low self-efficacy and high risk perception) (Figure 1). Demographic and clinical characteristics in four subgroups are presented in Table 1. To further elucidate specific group differences, *post-hoc* pairwise comparisons were conducted, and these detailed sociodemographic disparities are presented in Table 2.

**Table 1:** Sample characteristics and categorical variable univariate analysis of subgroups of coronary heart disease patients.

**Item**	**Total (*N* = 330)**	**Responsive (*****n*** = **22)**			**Proactive (*****n*** = **136)**			**Indifference (*****n*** = **27)**			**Avoidance (*****n*** = **145)**		
		**HISB**	**t/F/z/H**	***P***	**HISB**	**t/F/z/H**	***P***	**HISB**	**t/F/z/H**	***P***	**HISB**	**t/F/z/H**	***P***
Sex			2.034^(1)^	0.055		−3.378^(3)^	< 0.001^**^		−0.497^(3)^	0.619		−0.025^(3)^	0.980
Male	227 (68.5%)	17 (77.3)			97 (71.3)			21 (77.8)			92 (63.4)		
Female	103 (31.5%)	5 (22.7)			39 (28.7)			6 (22.2)			53 (36.6)		
Age			0.456^(2)^	0.640		11.901^(4)^	0.003^**^		0.443^(4)^	0.506		39.780^(4)^	< 0.001^**^
30–49	30(9.1%)	1 (4.5)			24 (17.6)			0 (0.00)			5 (3.4)		
50–69	181 (54.8%)	11 (50.0)			85 (62.5)			19 (70.4)			66 (45.5)		
≥70	119 (36.1%)	10 (45.5)			27 (19.9)			8 (29.6)			74 (51.0)		
Marital status			1.870^(2)^	0.181		4.734^(4)^	0.192		4.570^(4)^	0.102		2.190^(4)^	0.335
Unmarried	6 (1.8%)	1 (4.5)			3 (2.2)			1 (3.7)			1 (0.7)		
Married	204 (61.8%)	20 (90.9)			123 (90.4)			25 (92.6)			136(93.8)		
Divorced	0 (0.0%)	0 (0.00)			3 (2.2)			0 (0.00)			0 (0.00)		
Widowed	12 (3.6%)	1 (4.5)			7 (5.1)			1 (3.7)			8 (5.5)		
Education			1.919^(2)^	0.174		61.403^(4)^	< 0.001^**^		6.078^(4)^	0.193		35.202^(4)^	< 0.001^**^
Primary and below	146 (44.2%)	7 (31.8)			30 (22.1)			11 (40.7)			98 (67.6)		
Junior high school	87 (26.4%)	8 (36.4)			35 (25.7)			9 (33.3)			35 (24.1)		
High school/ Technical secondary school	56 (16.9%)	7 (31.8)			38 (27.9)			2 (7.4)			9 (6.2)		
Junior college	21 (6.4%)	0 (0.00)			18 (13.2)			2 (7.4)			1 (0.7)		
Bachelor degree and above	20 (6.1%)	0 (0.00)			15 (11.0)			3 (11.1)			2 (1.4)		
Occupation			4.063^(2)^	0.017^*^		52.796^(4)^	< 0.001^**^		16.520^(4)^	0.006^**^		30.367^(4)^	< 0.001^**^
Peasants	125 (37.9%)	7 (31.8)			23 (16.9)			2 (7.4)			93 (64.1)		
Workers	12 (3.6%)	3 (13.6)			1 (0.7)			1 (3.7)			7 (4.8)		
Civil servants	5 (1.5%)	0 (0.00)			4 (2.9)			0 (0.00)			1 (0.7)		
Utilities	17 (5.2%)	3 (13.6)			8 (5.9)			2 (7.4)			4 (2.8)		
Company Employee	18 (5.5%)	0 (0.00)			10 (7.4)			0 (0.00)			4 (2.8)		
Freelance	13 (3.9%)	2 (9.1)			7 (5.1)			1 (3.7)			3 (2.1)		
Retirees	127 (38.5%)	7 (31.8)			75 (55.1)			18 (66.7)			27 (18.6)		
Others	17 (5.2%)	0 (0.00)			8 (5.9)			3 (11.1)			6 (4.1)		
Per capita monthly household income			1.767^(2)^	0.177		31.789^(4)^	< 0.001^**^		10.390^(4)^	0.109		40.310^(4)^	< 0.001^**^
< 6,000	135 (40.9%)	7 (31.8)			33 (24.3)			3 (11.1)			92 (63.4)		
6,000–8,000	61 (18.5%)	4 (18.2)			32 (23.5)			5 (18.5)			20 (13.8)		
8,000–10,000	46 (13.9%)	1 (4.5)			21 (15.4)			4 (14.8)			20 (13.8)		
10,000-15,000	27 (8.2%)	4 (18.2)			16 (11.8)			5 (18.5)			2 (1.4)		
15,000–20,000	16 (4.8%)	0 (0.00)			11 (8.1)			3 (11.1)			2 (1.4)		
20,000–30,000	28 (8.5%)	5 (22.7)			13 (9.6)			5 (18.5)			5 (3.4)		
>30,000	22 (6.7%)	1 (4.5)			10 (7.4)			2 (7.4)			4 (2.8)		
Place of Residence			4.696^(2)^	0.022^*^		35.690^(4)^	< 0.001^**^		4.775^(4)^	0.092		27.576^(4)^	< 0.001^**^
Central urban area	132 (40.0%)	8 (36.4)			72 (52.9)			16 (59.3)			36 (24.8)		
Town or town-village area	47 (14.2%)	3 (13.6)			37 (27.2)			3 (11.1)			4 (2.8)		
Rural	70 (21.2%)	11 (50.0)			27 (19.9)			8 (29.6)			105(72.4)		
Type of medical insurance			3.129^(2)^	0.051		51.179^(4)^	< 0.001^**^		7.368^(4)^	0.025^*^		24.144^(4)^	< 0.001^**^
Urban employees'	94 (28.5%)	2 (9.1)			63 (46.3)			8 (29.6)			21 (14.5)		
Urban residents'	88 (26.7%)	8 (36.4)			44 (32.4)			10 (37.0)			26 (17.9)		
New rural cooperative	146 (44.2%)	11 (50.0)			28 (20.6)			9 (33.3)			98 (67.6)		
Commercial	1 (0.3%)	0 (0.00)			1 (0.7)			0 (0.00)			0 (0.00)		
Self-financed	1 (0.3%)	1 (4.5)			0 (0.00)			0 (0.00)			0 (0.00)		
Number of other diseases			1.279^(2)^	0.301		9.384^(4)^	0.009^**^		4.694^(4)^	0.096		2.151^(4)^	0.341
0	105 (31.8%)	8 (36.4)			52 (38.2)			6 (22.2)			39 (26.9)		
1	141 (42.7%)	7 (31.8)			53 (39.0)			10 (37.0)			71 (49.0)		
≥2	84 (25.5%)	7 (31.8)			31 (22.8)			11 (40.7)			35 (24.1)		
Time of diagnosis			3.240^(2)^	0.062		4.903^(4)^	0.179		3.994^(4)^	0.262		42.560^(4)^	< 0.001^**^
≤ 1year	179 (54.2%)	14 (63.6)			72 (52.9)			19 (70.4)			74 (51.0)		
1–5years	64 (19.4%)	5 (22.7)			27 (19.9)			6 (22.2)			26 (17.9)		
5–10years	68 (20.6%)	3 (13.6)			26 (19.1)			1 (3.7)			38 (26.2)		
≥10years	19 (5.8%)	0 (0.00)			11 (8.1)			1 (3.7)			7 (4.8)		
Whether PCI surgery			0.349^(1)^	0.731		−2.304^(3)^	0.021^*^		−2.662^(3)^	0.008^**^		−3.395^(3)^	< 0.001^**^
Yes	224 (67.9%)	16 (72.7)			95 (69.9)			21 (77.8)			92 (63.4)		
No	106 (32.1%)	6 (27.3)			41 (30.1)			6 (22.2)			53 (36.6)		
Number of stents			0.075^(2)^	0.973		12.522^(4)^	0.006^**^	12.213^(4)^	0.007^**^			37.479^(4)^	< 0.001^**^
0	106 (32.1%)	6 (27.3)			41 (30.1)			6 (22.2)			53 (36.6)		
1	62 (18.8%)	5 (22.7)			36 (26.5)			2 (7.4)			19 (13.1)		
2	57 (17.3%)	1 (4.5)			21 (15.4)			6 (22.2)			29 (20.0)		
≥3	47 (14.2%)	10 (45.5)			38 (27.9)			13 (48.1)			44 (30.3)		
Whether regular medication			NA	NA		−1.862^(3)^	0.063		−1.238^(3)^	0.216		−0.960^(3)^	0.337
Yes	312 (94.5%)	22 (100.00)			133 (97.8)			24 (88.9)			133(91.7)		
No	18 (5.5%)	0 (0.00)			3 (2.2)			3 (11.1)			12 (8.3)		
Amount of drugs taken			6.168^(2)^	0.005^**^		2.053^(4)^	0.561		2.369^(4)^	0.499		1.864^(4)^	0.601
0–2	50 (15.2%)	4 (18.2)			21 (15.4)			5 (18.5)			20 (13.8)		
3–5	187 (56.7%)	8 (36.4)			73 (53.7)			13 (48.1)			93 (64.1)		
6–8	73 (22.1%)	9 (40.9)			38 (27.9)			3 (11.1)			23 (15.9)		
≥9	16 (4.8%)	1 (4.5)			3 (2.2)			6 (22.2)			6 (4.1)		
Risk Perception		39.5 (37.00, 45.75)			20 (17.00, 23.00)			24 (20.00, 28.00)			44 (37.00,47.00)		
Physical diagnosis and treatment risk		19.36 ± 4.271			10 (9.00, 12.75)			10 (10.00, 14.00)			18 (17.00,20.00)		
Economic risk		16 (10.25, 20.00)			6 (4.00, 8.00)			7 (4.00, 9.00)			17 (16.00,20.00)		
Psychosocial risk		6.5 (6.00, 9.00)			3 (3.00, 4.00)			5 (3.00, 6.00)			7 (6.00,9.50)		
Self-efficacy		39 (37.00, 40.00)		45 (42.00, 346.00)			34 (31.00, 35.00)			31 (29.00,32.00)		

^*^p ≤ 0.05, ^**^p < 0.01.

HISB, Health Information Seeking Behavior; NA, Whether regular medication in the Responsive group was all Yes, and comparative analyses between groups were not possible.

(1) = Independent samples t-tests; (2) = One-way ANOVA; (3) = The Mann -Whitney U test; (4) = The Kruskal-Wallis H-test.

**Table 2:** *Post-hoc* analysis of univariate tests for categorical variables in each subgroup.

**Responsive (*****n*** = **22)**					
**Item**	**I-J**	**AD**	**SE**	***P***	**95% CI**
Occupation	Workers : Utilities	−41.000	12.036	0.024^(1)^	[−77.62, −4.38]
				0.034^(2)^	[−79.78, −2.22]
Place of residence	Central urban area : Rural	22.295	7.412	0.019^(1)^	[3.46, 41.13]
				0.022^(2)^	[2.84, 41.75]
Amount of drugs taken	NA	NA	NA	NA	NA
**Proactive (*****n*** = **136)**					
**Item**	**I-J**	**TS**	**SE**	***P***	
Age	≥70: 30–49	37.338	11.037	0.002^(2)^	
	50–69: 30–49	24.473	9.094	0.021^(2)^	
Education	Primary and below: Junior high school	−32.440	9.788	0.009^(2)^	
	Primary and below: High school/Technical secondary school	−54.441	9.608	< 0.001^(2)^	
	Primary and below: Junior college	−59.172	11.729	< 0.001^(2)^	
	Primary and below: Bachelor degree and above	−85.150	12.441	< 0.001^(2)^	
	Junior high school: Bachelor degree and above	−52.710	12.141	< 0.001^(2)^	
Occupation	Peasants: Retirees	−47.595	9.377	< 0.001^(2)^	
	Peasants: Utilities	−61.247	16.148	0.004^(2)^	
	Peasants: Freelance	−65.792	16.982	0.003^(2)^	
	Peasants: Company Employee	−78.735	14.902	< 0.001^(2)^	
	Peasants: Civil servants	−106.435	21.313	< 0.001^(2)^	
	Others: Civil servants	77.813	24.091	0.035^(2)^	
Per capita monthly household income	< 6,000: 8,000–10,000	−37.470	10.982	0.014^(2)^	
	< 6,000: 6,000–8,000	−38.782	9.761	0.001^(2)^	
	< 6,000: 20,000–30,000	−40.816	12.882	0.032^(2)^	
	< 6,000: 10,000–15,000	−48.688	11.985	0.001^(2)^	
	< 6,000: 15,000–20,000	−50.742	13.697	0.004^(2)^	
	< 6,000: >30,000	−52.020	14.201	0.005^(2)^	
Place of residence	Rural: Central urban area	49.859	8.878	< 0.001^(2)^	
	Rural: Town or town-village area	51.706	9.958	< 0.001^(2)^	
Type of medical insurance	New rural cooperative: Urban residents'	39.183	9.511	< 0.001^(2)^	
	New rural cooperative: Urban employees'	63.575	8.936	< 0.001^(2)^	
	Urban residents': Urban employees'	24.392	7.729	0.010^(2)^	
Number of other diseases	≥2: 0	25.319	8.927	0.014^(2)^	
Number of stents	0: ≥3	−23.382	8.859	0.050^(2)^	
**Indifference (*****n*** = **27)**					
**Item**	**I-J**	**TS**	**SE**	***P***	
Occupation	Peasants: Utilities	−23.250	7.913	0.050^(2)^	
Type of medical insurance	New rural cooperative: Urban employees'	9.528	3.845	0.040^(2)^	
Number of stents	2: 0	15.083	4.569	0.006^(2)^	
Age	≥70: 50–69	42.318	7.068	< 0.001^(2)^
	≥70: 30–49	57.650	19.291	0.008^(2)^
Education	Primary and below: Junior high school	−29.005	8.221	0.004^(2)^
	Primary and below: High school/Technical secondary school	−64.299	14.541	< 0.001^(2)^
Occupation	/	/	/	/
Per capita monthly household income	< 6,000: 6,000–8,000	−36.598	10.300	0.008^(2)^
	< 6,000: 8,000–10,000	−42.273	10.300	0.001^(2)^
	< 6,000: >30,000	−79.973	21.324	0.004^(2)^
Place of residence	Rural: Central urban area	36.306	8.063	< 0.001^(2)^
	Rural: Town or town-village area	66.473	21.268	0.005^(2)^
Type of medical insurance	New rural cooperative: Urban residents'	31.156	9.210	0.002^(2)^
	New rural cooperative: Urban employees'	41.641	10.039	< 0.001^(2)^
Time of diagnosis	5–10years : ≤ 1years	43.823	8.332	< 0.001^(2)^
	5–10years : ≤ 1–5years	63.283	10.626	< 0.001^(2)^
Number of stents	≥3: 1	36.238	11.461	0.009^(2)^
	≥3: 0	47.169	8.515	5.540^(2)^
	≥3: 2	48.349	9.986	< 0.001^(2)^

AD, Average Difference; SE, Standard Error; TS, Test Statistics; NA, *Post-hoc* tests will not be performed for Health Information Seeking Behavior due to at least one group having fewer than two cases.

/ = *p* >0.05.

(1) = Tukey HSD; (2) = Bonferroni.

### Differences in HISB across subgroups

**Figure 2:**
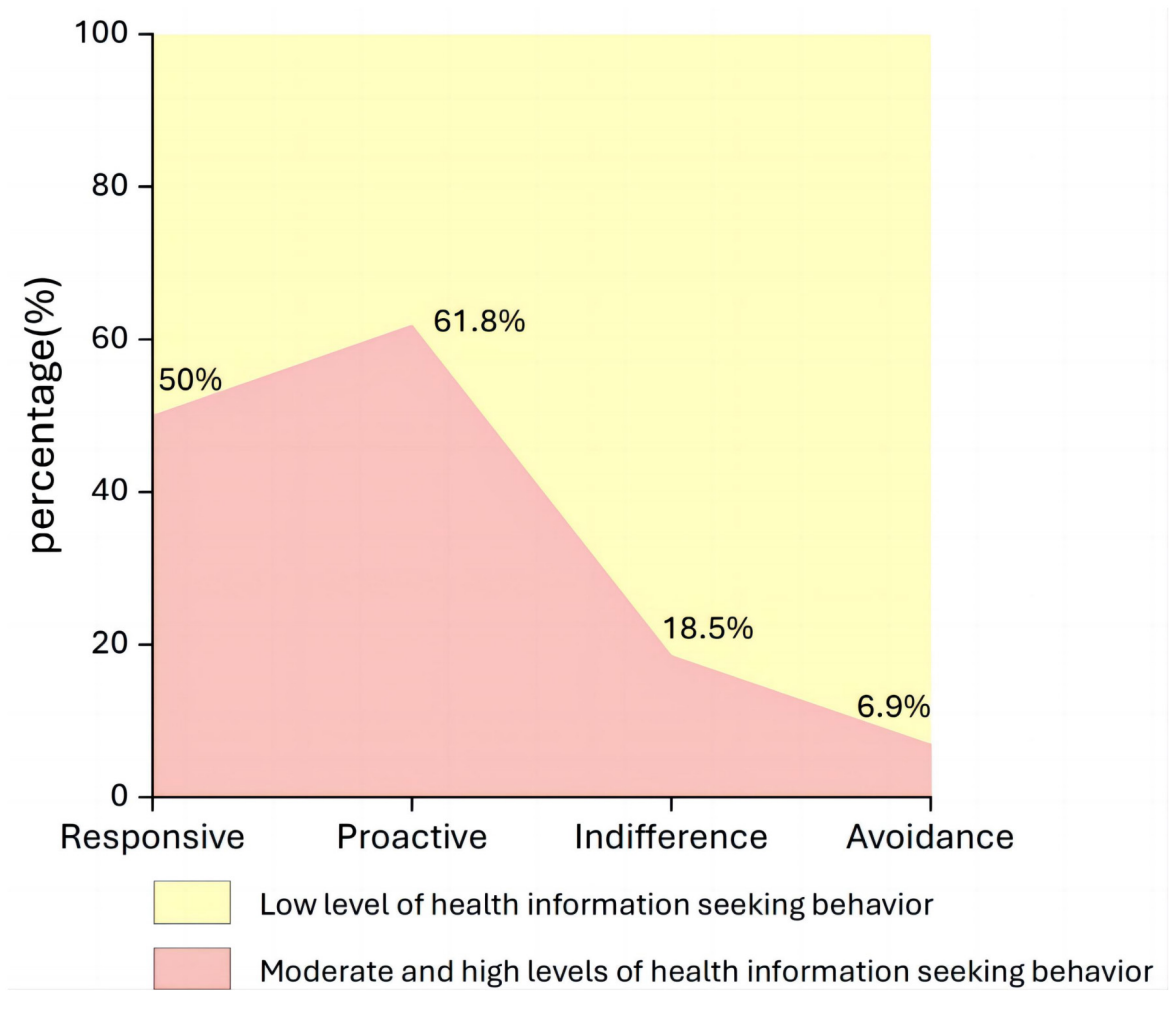
Distribution of HISB levels across the four RPA subgroups. The area chart displays the proportion of patients within each subgroup classified as having low HISB (total score < 78, indicated in light yellow) vs. moderate-to-high HISB (total score ≥ 78, indicated in pink). The cut-off point of 78 corresponds to the sample mean. Percentages indicate the proportion of patients with moderate-to-high HISB in each group.

The distributional characteristics of HISB in the four subgroups were further analyzed, and the level of HISB of patients was measured by the total score (ranging from 26 to 130). Consistent with established methodologies in RPA research (12, 15), which rely on sample distributions to define relative categories, the sample mean (78.00) was utilized as the empirical cut-off point. Scores ≥78 were classified as moderate-to-high levels of HISB, and scores < 78 were classified as low levels of HISB (Figure 2). HISB varied significantly across the four subgroups of coronary heart disease patients. Responsive patients were almost equally split above and below the sample mean, whereas 61.8 % of Proactive patients scored ≥78. In contrast, 81.5% of Indifferent and 93.1% of Avoidance patients fell below the mean, indicating predominantly low HISB.

### Factors associated with HISB in each subgroup

Variables that exhibited univariate associations in Tables 1 and 3 were entered into the multivariable models. Among Responsive patients, only physical diagnosis and treatment risk (β = 2.049, 95%CI (0.528, 3.570)] remained independently linked to higher HISB. For Proactive participants, education [β = 4.725, 95%CI (2.272, 7.178)], per capita monthly household income [β = 1.680, 95%CI (0.177, 3.183)], self-efficacy [β = 0.927, 95%CI (0.011, 1.843)] were independently associated with increased HISB, whereas economic risk [β = −1.654, 95%CI (−3.214, −0.094)], type of medical insurance [β = −5.814, 95%CI (−8.800, −2.828)], number of other diseases [β = −3.651, 95%CI (−6.560, −0.742)] were independently associated with decreased HISB. In Indifferent patients, the type of medical insurance [β = −6.447, 95%CI (−12.503, −0.391)] was the only significant factor correlated with lower HISB. Among Avoidance patients, older age [β = −4.757, 95%CI (−8.525, −0.989)] was independently associated with lower HISB, while education [β = 5.432, 95%CI (2.353, 8.511)] was independently associated with higher HISB (Table 4).

**Table 3:** Continuous variable correlation analysis of subgroups of coronary heart disease patients.

**Variant**	**HISB**	**Self-efficacy**	**Risk perception**	**Economic risk**	**Physical diagnosis and treatment risk**	**Psychosocial risk**
HISB	1					
Self−efficacy	^a^−0.023 ^b^0.421^**^ ^c^0.280 ^d^0.022	1				
Risk perception	^a^−0.128 ^b^−0.230^**^ ^c^−0.281 ^d^−0.431^**^	^a^0.192 ^b^−0.234^**^ ^c^0.105 ^d^0.209^*^	1			
Economic risk	^a^−0.563^**^ ^b^−0.456^**^ ^c^−0.158 ^d^−0.518^**^	^a^0.304 ^b^−0.274^**^ ^c^−0.089 ^d^0.183^*^	^a^0.735^**^ ^b^0.662^**^ ^c^0.460^*^ ^d^0.829^**^	1		
Physical diagnosis and treatment risk	^a^0.468^*^ ^b^0.014 ^c^−0.164 ^d^0.001	^a^0.056 ^b^−0.046 ^c^0.223 ^d^−0.080	^a^0.664^**^ ^b^0.878^**^ ^c^0.840^**^ ^d^0.421^**^	^a^0.021 ^b^0.330^**^ ^c^0.009 ^d^0.132	1	
Psychosocial risk	^a^−0.149 ^b^−0.096 ^c^0.156 ^d^−0.295^**^	^a^−0.327 ^b^−0.066 ^c^−0.020 ^d^0.259^**^	^a^0.541^**^ ^b^0.191^*^ ^c^0.169 ^d^0.578^**^	^a^0.344 ^b^−0.034 ^c^−0.054 ^d^0.320^**^	^a^0.378 ^b^0.074 ^c^0.113 ^d^−0.132	1

^*^p ≤ 0.05, ^**^p < 0.01.

^a^Responsive, ^b^Proactive, ^c^Indifference, ^d^Avoidance.

**Table 4:** Multifactorial analysis of characteristics of subgroups of patients with coronary heart disease.

**Characteristics of subgroups**	**Unstandardized coefficient**		**Standardized coefficient**	***t***	***P***	**Collinear statistics**	
	***B***	**SE**	β			**Tolerances**	**VIF**
**Responsive**							
(Constant)	72.813	33.429		2.178	0.045^*^		
Economic risk	−1.279	0.979	−0.320	−1.306	0.210	0.380	2.633
Physical diagnosis and treatment risk	2.049	0.774	0.472	2.647	0.018^*^	0.719	1.391
Occupation	−0.841	1.229	−0.159	−0.685	0.503	0.421	2.373
Place of residence	−8.719	5.437	−0.442	−1.604	0.128	0.300	3.332
Amount of drugs taken	1.260	4.553	0.057	0.277	0.786	0.539	1.857
**Proactive**							
(Constant)	38.585	24.445		1.578	0.117		
Economic risk	−1.654	0.796	−0.182	−2.078	0.040^*^	0.391	2.559
Self-efficacy	0.927	0.467	0.137	1.983	0.050^*^	0.633	1.581
Risk perception	0.309	0.321	0.077	0.963	0.337	0.470	2.127
Sex	−0.214	2.672	−0.005	−0.080	0.936	0.686	1.457
Whether PCI surgery	3.889	4.122	0.098	0.943	0.347	0.280	3.572
Age	−3.119	1.909	−0.104	−1.634	0.105	0.734	1.362
Education	4.725	1.249	0.327	3.783	< 0.001^**^	0.403	2.481
Occupation	−0.227	0.392	−0.040	−0.579	0.564	0.637	1.569
Per capita monthly household income	1.680	0.763	0.176	2.201	0.030^*^	0.470	2.130
Place of residence	2.783	2.316	0.120	1.201	0.232	0.302	3.313
Type of medical insurance	−5.814	1.521	−0.377	−3.823	< 0.001^**^	0.309	3.241
Number of other diseases	−3.651	1.484	−0.153	−2.461	0.015^*^	0.776	1.288
Number of stents	1.453	1.509	0.094	0.963	0.337	0.313	3.191
**Indifference**							
(Constant)	4.124	39.354		0.105	0.917		
Whether PCI surgery	37.089	18.428	0.735	2.013	0.057	0.216	4.635
Occupation	1.022	1.340	0.131	0.763	0.453	0.972	1.029
Type of medical insurance	−6.447	3.075	−0.376	−2.096	0.048^*^	0.897	1.115
Number of stents	11.124	6.266	0.637	1.775	0.090	0.224	4.474
**Avoidance**							
(Constant)	72.162	11.781		6.126	< 0.001^**^		
Economic risk	−0.577	0.456	−0.177	−1.265	0.208	0.204	4.903
Psychosocial risk	0.391	0.468	0.076	0.837	0.404	0.488	2.050
Risk perception	−0.237	0.297	−0.115	−0.798	0.426	0.194	5.151
Whether PCI surgery	2.306	3.914	0.096	0.589	0.557	0.151	6.608
Age	−4.757	1.916	−0.232	−2.483	0.014^*^	0.460	2.174
Education	5.432	1.565	0.356	3.471	< 0.001^**^	0.380	2.632
Occupation	−0.667	0.395	−0.197	−1.688	0.094	0.293	3.418
Per capita monthly household income	1.222	0.753	0.155	1.622	0.107	0.437	2.289
Place of residence	−0.594	1.876	−0.044	−0.317	0.752	0.205	4.882
Type of medical insurance	0.807	1.780	0.082	0.453	0.651	0.122	8.205
Time of diagnosis	−1.177	0.977	−0.099	−1.204	0.231	0.597	1.674
Number of stents	0.138	1.574	0.015	0.087	0.930	0.137	7.299

^*^p ≤ 0.05, ^**^p < 0.01.

SE, Standard error; β, Beta.

## Discussion

In this study, we employed the RPA framework to classify 330 CHD patients into four subgroups, each characterized by a unique combination of self-efficacy and risk perception.

The Responsive subgroup, characterized by high levels of both risk perception and self-efficacy. In this group, the perceived risk of physical diagnosis and treatment was identified as the most significant factor associated with higher HISB. Interpreted through the RPA framework, this strong positive association suggested that this group was both risk-sensitive and capable of translating perceived risk into action; their high self-efficacy allowed them to confront potential physical threats via proactive seeking rather than avoidance (14, 18). However, *post-hoc* analysis highlighted significant disparities driven by external resources within this active group. Specifically, residence in central urban areas and employment in utilities (compared to rural living and manual labor) were associated with significantly higher HISB. This indicated that even with high motivation, structural barriers—such as the digital divide in rural areas or rigid work schedules for laborers—could impede information access (19, 20). Consequently, interventions had to focus on leveraging digital equity to bridge these resource gaps. Healthcare providers had to implement mobile-optimized telemedicine platforms and asynchronous consultation systems to bypass geographic and time constraints. For rural or working-class patients, AI-assisted diagnostic interpretation tools could be deployed via smartphones, allowing them to instantly access clinical-grade explanations of their physical symptoms without requiring travel to urban centers, thus ensuring their proactive nature was supported by equitable resource allocation (21, 22).

The Proactive subgroup was characterized by low-risk perception and high self-efficacy. Within this group, Economic risk, Self-efficacy, Education, Per capita monthly household income, and Type of medical insurance were identified as significant factors of increased HISB. Theoretically, these individuals did not feel immediately threatened but believed in their ability to stay healthy, motivating them to engage in HISB to optimize their wellbeing (6). However, *post-hoc* analysis revealed a significant socioeconomic divide, where low socioeconomic status individuals (characterized by primary education, income < 6,000, and New Rural Cooperative Medical Scheme coverage) exhibited distinct behavioral patterns positively driven by Economic Risk. Therefore, management strategies had to prioritize economic framing by moderately increasing their perception of economic risk to catalyze engagement (23, 24). Therefore, the Healthcare Security Administration had to explicitly publicize the “reimbursement ceilings” and non-covered costs of rural insurance to correct their underestimated risk perception. By framing the potential for “poverty due to illness” as a tangible threat, this strategy directed their high self-efficacy toward preventative information seeking as a vital skill for household economic protection (25–27).

The Indifference subgroup presented the lowest levels of HISB, a state of behavioral inertia characterized by both low-risk perception and low self-efficacy. Within this group, Type of medical insurance was identified as a significant factor negatively associated with HISB. Theoretically, the combination of low-risk perception and low self-efficacy suggests a state of passivity where individuals lack the motivation to engage (6). *Post-hoc* analysis regarding stent counts further reinforces this, suggesting that without the tangible burden of severe disease, these patients perceive no immediate necessity to inquire, resulting in deep passivity. To break this cycle of indifference, interventions must shift from passive availability to incentivized engagement (28). Specifically, it is suggested that community hospitals organize regular health education lectures that are strictly bundled with “routine examinations within the insurance scope.” By linking information delivery to the tangible benefit of covered check-ups (e.g., inviting them for a free blood glucose or lipid test available under their specific insurance), medical staff can effectively draw these passive patients into the management system (29).

The Avoidance subgroup, characterized by high-risk perception but low self-efficacy, exhibited predominantly low HISB, with older age and lower education identified as independent factors associated with information avoidance. In the context of the Extended Parallel Process Model (EPPM), these patients were likely trapped in “fear control,” where the overwhelming threat of the disease combined with a lack of confidence led to defensive avoidance rather than adaptive action (30). *Post-hoc* analysis revealed a “Capacity-Pressure” divergence: lower socioeconomic factors (primary and below education, income < 6,000) were linked to lower HISB, while factors reflecting accumulated disease burden (age ≥70, diagnosis 5–10 years, stents ≥3) were associated with higher HISB. This suggested that for the Avoidance group, the main barrier was not a lack of need, but cognitive and financial “thresholds” to information seeking. Therefore, instead of overwhelming these patients with complex online information, clinicians should utilize traditional, user-friendly formats such as illustrated printed brochures, telephone counseling, and small-group education sessions (31). Additionally, involving family members as information proxies could provide the necessary social scaffolding to reduce anxiety and gradually enhance the patient's confidence in managing their disease information.

## Limitations

First, the study used convenience sampling from a single tertiary hospital, limiting generalizability. Participants may overrepresent those with higher disease severity or better access to medical resources, affecting external validity. Second, the cross-sectional design restricts causal inference, and self-reported data may introduce bias. Third, the small sample sizes in the “Responsive” and “Indifference” subgroups limit statistical power and model stability, although they reflect the inherent distribution of RPA profiles. Future research should use larger, more diverse samples, longitudinal designs, and objective measures to validate findings, especially for these smaller subgroups.

## Conclusions

Each group's unique characteristics and associations with HISB underscore the need for tailored approaches. Understanding the motivations and barriers specific to each group allows for more effective and personalized interventions, enhancing health outcomes for patients with coronary heart disease. Future research could consider developing and testing tailored health information interventions for different patient groups. Additionally, leveraging technological platforms, such as mobile health applications and online communities, could provide patients with more convenient and personalized health information and support. These technologies could also assist in collecting more accurate data on HISB and provide real-time feedback to patients.

## Data Availability

The raw data supporting the conclusions of this article will be made available by the authors, without undue reservation.

## References

[1] Zhao K Shen X Liu H Lin Z Li J Chen S . Somatic and germline variants and coronary heart disease in a Chinese population. JAMA Cardiol. (2024) 9:233–42. doi: 10.1001/jamacardio.2023.5095 38198131 PMC10782380

[2] Yan W Qingyin H. Latent profile analysis of health information acquisition behaviors in patients with newly diagnosed type 2 diabetes mellitus and its influencing factors. Chin Nurs Manag. (2022) 22:1782–6. doi: 10.3969/j.issn.1672-1756.2022.12.006

[3] Zhu Y Song Y Wang Y Ji H Wang D Cai S . Relationships among patient activation, social support and online health information seeking of community-dwelling older adults living with coronary heart disease. J Adv Nurs. (2023) 79:161–9. doi: 10.1111/jan.15428 36052639

[4] Svavarsdóttir MH Halapi E Ketilsdóttir A Ólafsdóttir IV Ingadottir B. Changes in disease-related knowledge and educational needs of patients with coronary heart disease over a six-month period between hospital discharge and follow-up. Patient Educ Couns. (2023) 117:107972. doi: 10.1016/j.pec.2023.107972 37703621

[5] Yaru Z. Study on the status and influencing factors of online health information seeking behavior in older patients with coronary heart disease. [Master Thesis]. Qingdao University, Qingdao, China (2023).

[6] Mirzaei A Aslani P Luca EJ Schneider CR. Predictors of health information-seeking behavior: systematic literature review and network analysis. J Med Internet Res. (2021) 23:e21680. doi: 10.2196/21680 33979776 PMC8285748

[7] Bae SY Chang P-J. The effect of coronavirus disease-19 (COVID-19) risk perception on behavioural intention towards ‘untact' tourism in south korea during the first wave of the pandemic (March 2020). Curr Issues Tour. (2021) 24:1017–35. doi: 10.1080/13683500.2020.1798895

[8] Huan L Jina Z Yunxia M Qinglin W Xiaochuang L. Risk perception of cardiovascular disease and its impact on self-management in rural elderly patients with hypertension. J Nurs Sci. (2023) 38:12–5, 33. doi: 10.3870/j.issn.1001-4152.2023.19.012

[9] Purcell H Kohler IV Ciancio A Mwera J Delavande A Mwapasa V . Mortality risk information and health-seeking behavior during an epidemic. PNAS. (2024) 121:e2315677121. doi: 10.1073/pnas.2315677121 38959039 PMC11252761

[10] Li J Ye Z Zhuang J Okada N Huang L Han G . Changes of public risk perception in China: 2008–2018. Sci Total Environ. (2021) 799:149453. doi: 10.1016/j.scitotenv.2021.149453 34388887

[11] Bandura A. Self-efficacy. In:Ramachandran VS, editor. Encyclopedia of Human Behavior, Vol. 4. New York: Academic Press (1994). p. 71–81. (Reprinted in H. Friedman [Ed.], *Encyclopedia of mental health*. San Diego: Academic Press (1998).

[12] Rimal RN Juon H-S. Use of the risk perception attitude framework for promoting breast cancer prevention. J Appl Soc Psychol. (2010) 40:287–310. doi: 10.1111/j.1559-1816.2009.00574.x

[13] Kim M-S Kim S-H. Health information-seeking behavior in patients with coronary artery disease: activating methods. PLoS ONE. (2024) 19:e0300755. doi: 10.1371/journal.pone.0300755 38630654 PMC11023259

[14] Rimal RN Real K. Perceived risk and efficacy beliefs as motivators of change. Hum Commun Res. (2003) 29:370–99. doi: 10.1111/j.1468-2958.2003.tb00844.x

[15] Grasso KL Bell RA. Understanding health information seeking: a test of the risk perception attitude framework. J Health Commun. (2015) 20:861–70. doi: 10.1080/10810730.2015.1018634 26161622

[16] Lei F Panyu R Yinling Z Baohua C. Primary development of risk perception questionnaire for chronic patients. Chin J Health Psychol. (2014) 1865–7. doi: 10.13342/j.cnki.cjhp.2014.12.039

[17] Xuemei W Jianwei Z Yuting X Fei C Jun L Fang L. Translation of the heart health self-efficacy and self-management scale and testing its psychometric properties. Chin Nurs Manag. (2022) 22:852–7. doi: 10.3969/j.issn.1672-1756.2022.06.011

[18] Kanna B Ukudeyeva A Faiz M Roques E Washington T Ramirez L . Qualitative study of knowledge, perception, behavior and barriers associated with cardiovascular disease risk among overweight and obese Hispanic taxi drivers of south Bronx, NYC. BMC Public Health. (2020) 20:683. doi: 10.1186/s12889-020-08751-0 32410613 PMC7222470

[19] Wang X Shi J Lee KM. The digital divide and seeking health information on smartphones in Asia: survey study of ten countries. J Med Internet Res. (2022) 24:e24086. doi: 10.2196/24086 35023845 PMC8796039

[20] Eruchalu CN Pichardo MS Bharadwaj M Rodriguez CB Rodriguez JA Bergmark RW . The expanding digital divide: digital health access inequities during the COVID-19 pandemic in New York City. J Urban Health. (2021) 98:183–6. doi: 10.1007/s11524-020-00508-9 33471281 PMC7816740

[21] Funer F Schneider D Heyen NB Aichinger H Klausen AD Tinnemeyer S . Impacts of clinical decision support systems on the relationship, communication, and shared decision-making between health care professionals and patients: multistakeholder interview study. J Med Internet Res. (2024) 26:e55717. doi: 10.2196/55717 39178023 PMC11380058

[22] Vromans RD Tillier CN Pauws SC van der Poel HG van de Poll-Franse LV Krahmer EJ . Communication, perception, and use of personalized side-effect risks in prostate cancer treatment-decision making: an observational and interview study. Patient Educ Couns. (2022) 105:2731–9. doi: 10.1016/j.pec.2022.04.017 35534301

[23] Gallifant J Celi LA Pierce RL. Digital determinants of health: opportunities and risks amidst health inequities. Nat Rev Nephrol. (2023) 19:749–50. doi: 10.1038/s41581-023-00763-4 37626271

[24] Tang C Wu X Chen X Pan B Yang X. Examining income-related inequality in health literacy and health-information seeking among urban population in China. BMC Public Health. (2019) 19:1–9. doi: 10.1186/s12889-019-6538-2 30791882 PMC6385413

[25] Adams A Kluender R Mahoney N Wang J Wong F Yin W . The impact of financial assistance programs on health care utilization: evidence from Kaiser permanente. Am Econ Rev Insights. (2022) 4:389–407. doi: 10.1257/aeri.20210515 36338144 PMC9634821

[26] Ito T Kounnavong S Miyoshi C. Financial burden and health-seeking behaviors related to chronic diseases under the national health insurance scheme in bolikhamxay province, lao PDR: a cross-sectional study. Int J Equity Health. (2022) 21:180. doi: 10.1186/s12939-022-01788-0 36527068 PMC9758772

[27] Jia X Pang Y Liu LS. Online health information seeking behavior: a systematic review. Healthcare. (2021) 9:1740. doi: 10.3390/healthcare9121740 34946466 PMC8701665

[28] Lu K Xiong X Horras A Jiang B Li M. Impact of financial barriers on health status, healthcare utilisation and economic burden among individuals with cognitive impairment: a national cross-sectional survey. BMJ Open. (2022) 12:e056466. doi: 10.1136/bmjopen-2021-056466 35508339 PMC9073389

[29] Lee D-C Wang J Shi L Wu C Sun G. Health insurance coverage and access to care in China. BMC Health Serv Res. (2022) 22:140. doi: 10.1186/s12913-022-07498-1 35114992 PMC8812221

[30] Rezaei Aghuei A Hojjati H Hekmati Pour N Nejat H Akbari A. The effect of the extended parallel process model on self-efficacy of spouses of veterans with post-traumatic stress disorder. J Health Rep Technol. (2024) 10:e143160. doi: 10.5812/jhrt-143160

[31] Gordon NP Hornbrook MC. Older adults' readiness to engage with eHealth patient education and self-care resources: a cross-sectional survey. BMC Health Serv Res. (2018) 18:220. doi: 10.1186/s12913-018-2986-0 29587721 PMC5872546

