# Supplementary material for: From awareness to action: exploring health information seeking behavior in coronary heart disease patients: a cross-sectional study
Source: Front Public Health. 2026 Jan 27;14:1749036. doi: 10.3389/fpubh.2026.1749036 (PMC12886365; doi:10.3389/fpubh.2026.1749036)

**Supplementary Material**

**Supplementary File 1**

**Health Information Seeking Behavior Questionnaire for Chronic Disease Patients**

*Introduction: The following is a survey about health information seeking behavior. Please select the level that best describes your experience based on your past experiences in seeking health information.*

**Section 1: Health Information Needs**

*Please indicate how much you need the following types of health information (1 = Not needed at all, 5 = Always needed)*

| Item | Not needed at all | Rarely needed | Sometimes needed | Often needed | Always needed |
| --- | --- | --- | --- | --- | --- |
| 1. Basic disease knowledge (e.g., common symptoms, complications, heritability, health risks) | 1 | 2 | 3 | 4 | 5 |
| 2. Disease treatment methods (e.g., types of treatments, specific processes, outcomes, and prognosis) | 1 | 2 | 3 | 4 | 5 |
| 3. Diagnostic tests (e.g., common tests, purposes, interpreting results) | 1 | 2 | 3 | 4 | 5 |
| 4. Medication knowledge (e.g., drug effects, side effects, precautions) | 1 | 2 | 3 | 4 | 5 |
| 5. Risk factor management (e.g., identifying and controlling risk factors) | 1 | 2 | 3 | 4 | 5 |
| 6. Symptom management (e.g., monitoring, recognizing, and managing symptoms) | 1 | 2 | 3 | 4 | 5 |
| 7. Prevention and management of complications (e.g., recognizing, preventing, and handling complications) | 1 | 2 | 3 | 4 | 5 |
| 8. Diet and nutrition (e.g., recommended nutrients, dietary do's and don'ts) | 1 | 2 | 3 | 4 | 5 |
| 9. Exercise and physical activity (e.g., types of exercise, precautions such as duration and intensity) | 1 | 2 | 3 | 4 | 5 |
| 10. Work and daily life impact (e.g., how the disease affects daily activities and work) | 1 | 2 | 3 | 4 | 5 |
| 11. Psychological management (e.g., coping with negative emotions related to the disease) | 1 | 2 | 3 | 4 | 5 |
| 12. Professional medical support (e.g., types of support available, how to access it) | 1 | 2 | 3 | 4 | 5 |
| 13. Social support (e.g., who can help, how to get help) | 1 | 2 | 3 | 4 | 5 |

| **Section 2: Health Information Seeking Channels** *(for descriptive analysis only)*  *<1>Please indicate how often you have* ***actively*** *and* ***passively*** *used the following channels to seek health information (1 = Never, 5 = Always)* | | | | | |
| --- | --- | --- | --- | --- | --- |
| 14.How often you have **actively** used the following channels to seek health information | Never | Rarely | Sometimes | Often | Always |
| Newspapers, magazines, books | 1 | 2 | 3 | 4 | 5 |
| Television, radio, or podcasts | 1 | 2 | 3 | 4 | 5 |
| Health brochures | 1 | 2 | 3 | 4 | 5 |
| Internet | 1 | 2 | 3 | 4 | 5 |
| Healthcare professionals | 1 | 2 | 3 | 4 | 5 |
| Family and friends | 1 | 2 | 3 | 4 | 5 |
| Fellow patients | 1 | 2 | 3 | 4 | 5 |
| Other (please specify) | 1 | 2 | 3 | 4 | 5 |
| Other channels to **actively** seek health information: ___________________ (open-ended question) | | | | | |
| 15.How often you have **passively** used the following channels to seek health information | Never | Rarely | Sometimes | Often | Always |
| Newspapers, magazines, books | 1 | 2 | 3 | 4 | 5 |
| Television, radio, or podcasts | 1 | 2 | 3 | 4 | 5 |
| Health brochures | 1 | 2 | 3 | 4 | 5 |
| Internet | 1 | 2 | 3 | 4 | 5 |
| Healthcare professionals | 1 | 2 | 3 | 4 | 5 |
| Family and friends | 1 | 2 | 3 | 4 | 5 |
| Fellow patients | 1 | 2 | 3 | 4 | 5 |
| Other (please specify) | 1 | 2 | 3 | 4 | 5 |
| Other channels to **passively** seek health information: __________________(open-ended question) | | | | | |

*<2>Please indicate how often you have* ***actively*** *and* ***passively*** *used the following* ***online*** *channels to seek health information (1 = Never, 5 = Always)*

| 16.How often you have **actively** used the following **online** channels to seek health information | Never | Rarely | Sometimes | Often | Always |
| --- | --- | --- | --- | --- | --- |
| Search engines (Baidu, Sogou, 360 Browser, etc.) | 1 | 2 | 3 | 4 | 5 |
| Social media (WeChat, Weibo, TikTok, etc.) | 1 | 2 | 3 | 4 | 5 |
| Knowledge Q&A websites (Zhihu, Baidu Tieba, etc.) | 1 | 2 | 3 | 4 | 5 |
| Official websites of medical institutions and health administration departments | 1 | 2 | 3 | 4 | 5 |
| Health and medical websites or apps (Haodf.com, 39 Health, etc.) | 1 | 2 | 3 | 4 | 5 |
| Academic databases (CNKI, Wanfang, etc.) | 1 | 2 | 3 | 4 | 5 |
| Other | 1 | 2 | 3 | 4 | 5 |
| Other ***online*** channels to **actively** seek health information: ___________________ (open-ended question) | | | | | |
| 17.How often you have ***passively*** used the following **online** channels to seek health information | Never | Rarely | Sometimes | Often | Always |
| Social media (WeChat, Weibo, TikTok, etc.) | 1 | 2 | 3 | 4 | 5 |
| Knowledge Q&A websites (Zhihu, Baidu Tieba, etc.) | 1 | 2 | 3 | 4 | 5 |
| Official websites of medical institutions and health administration departments | 1 | 2 | 3 | 4 | 5 |
| Health and medical websites or apps (Haodf.com, 39 Health, etc.) | 1 | 2 | 3 | 4 | 5 |
| Academic databases (CNKI, Wanfang, etc.) | 1 | 2 | 3 | 4 | 5 |
| Other ***online*** channels to ***passively*** seek health information: ___________________ (open-ended question) | | | | | |

**Section 3: Health Information Seeking Abilities**

*Please indicate your level of agreement with the following statements (1 = Strongly disagree, 5 = Strongly agree)*

| “When I go looking for health-related information, ……” | Strongly disagree | Disagree | Neutral | Agree | Strongly agree |
| --- | --- | --- | --- | --- | --- |
| Before seeking information | | | | | |
| 18. I can recognize my health issues and needs | 1 | 2 | 3 | 4 | 5 |
| 19. I can clearly express my health information needs | 1 | 2 | 3 | 4 | 5 |
| While seeking information | | | | | |
| 20. I know where to find health-related information | 1 | 2 | 3 | 4 | 5 |
| 21. I can choose appropriate channels or methods to obtain health information based on my situation | 1 | 2 | 3 | 4 | 5 |
| When the results are not satisfactory | | | | | |
| 22. I can supplement health information through multiple channels | 1 | 2 | 3 | 4 | 5 |
| 23. I will persist until I find the information I need | 1 | 2 | 3 | 4 | 5 |

**Section 4: Health Information Evaluation**

*Please indicate your level of agreement with the following statements (1 = Strongly disagree, 5 = Strongly agree)*

| “For the health information obtained, ……” | Strongly disagree | Disagree | Neutral | Agree | Strongly agree |
| --- | --- | --- | --- | --- | --- |
| 24. I can understand the health information I obtain | 1 | 2 | 3 | 4 | 5 |
| 25. I can judge whether the health information is correct | 1 | 2 | 3 | 4 | 5 |
| 26. I can judge whether the health information meets my actual situation and needs | 1 | 2 | 3 | 4 | 5 |
| 27. I can judge whether the health information can be applied in daily life | 1 | 2 | 3 | 4 | 5 |
| 28. I compare the time and cost spent on different information sources | 1 | 2 | 3 | 4 | 5 |
| 29. I can judge the reliability of the information source | 1 | 2 | 3 | 4 | 5 |
| 30. I can judge the security of different information channels | 1 | 2 | 3 | 4 | 5 |

**Supplementary File 2**

**The Risk Perception Questionnaire for Chronic Disease Patients**

*Introduction: The following questions aim to assess your perception of risks associated with your treatment and health management. Please indicate how worried you are about each of the following statements. Your responses will help us understand your concerns and perceptions related to your health and treatment.*

| Item | Very unworried | Not Worried | Neutral | Worried | Very worried |
| --- | --- | --- | --- | --- | --- |
| 1. Treatment will increase the financial burden on myself and my family | 1 | 2 | 3 | 4 | 5 |
| 2. The current treatment plan is too expensive | 1 | 2 | 3 | 4 | 5 |
| 3. Spending money without achieving the expected treatment outcomes | 1 | 2 | 3 | 4 | 5 |
| 4. Treatment costs cause significant financial pressure | 1 | 2 | 3 | 4 | 5 |
| 5. Complications may arise during the treatment process | 1 | 2 | 3 | 4 | 5 |
| 6. Side effects of treatment may harm my health | 1 | 2 | 3 | 4 | 5 |
| 7. Medications are ineffective or not effective enough for my condition | 1 | 2 | 3 | 4 | 5 |
| 8. Diagnostic and therapeutic measures (special examinations, treatment procedures) may harm my body | 1 | 2 | 3 | 4 | 5 |
| 9. Errors may occur during treatment (e.g., wrong injections, wrong medications) | 1 | 2 | 3 | 4 | 5 |
| 10. Fear that healthcare providers will not respect or insult me | 1 | 2 | 3 | 4 | 5 |
| 11. The hospital environment and atmosphere will affect my psychological state | 1 | 2 | 3 | 4 | 5 |
| 12. Family members may experience burnout from long-term caregiving | 1 | 2 | 3 | 4 | 5 |

**Supplementary File 3**

**The Heart Health Self-Efficacy Scale**

*Introduction: The following questions are designed to assess your confidence and frequency in performing behaviors related to heart health. Please indicate how confident you are in your ability to perform each behavior and how often you perform it.*

| **Item** | **How confident are you that you can do the following?** | | | | **How often do you do the following?** | | | |
| --- | --- | --- | --- | --- | --- | --- | --- | --- |
|  | **Not confident** | **A little confident** | **Confident** | **Very confident** | **Rarely** | **sometimes** | **Often** | **Always** |
| 1.Engage in as much physical activity as possible every day  (e.g., reducing time spent sitting in front of the TV or computer, taking the stairs instead of the elevator) | 1 | 2 | 3 | 4 | 1 | 2 | 3 | 4 |
| 2.Perform at least 30 minutes of moderate-intensity physical activity on most days of the week  (e.g., walking, gardening, light housework) | 1 | 2 | 3 | 4 | 1 | 2 | 3 | 4 |
| 3.Reduce intake of saturated fats and trans fats  (e.g., fatty meats, butter and store-bought pies, pastries, biscuits) | 1 | 2 | 3 | 4 | 1 | 2 | 3 | 4 |
| 4.Minimize salt intake  (e.g., avoid high-salt foods like chips, soy sauce, takeout meals, and reduce salt used in cooking) | 1 | 2 | 3 | 4 | 1 | 2 | 3 | 4 |
| 5.Eat 2 servings of fruit daily  (140-200g, e.g., 1 apple; 1 cup of diced fruit or canned fruit) | 1 | 2 | 3 | 4 | 1 | 2 | 3 | 4 |
| 6.Eat 5 servings of vegetables daily  (350-500g, e.g., 1/2 cup of cooked vegetables or 1/2 of a medium-sized potato) | 1 | 2 | 3 | 4 | 1 | 2 | 3 | 4 |
| 7.Take medication as prescribed by your doctor | 1 | 2 | 3 | 4 | 1 | 2 | 3 | 4 |
| 8.Buy medication on time according to the prescription to prevent running out of medication | 1 | 2 | 3 | 4 | 1 | 2 | 3 | 4 |
| 9.Regularly visit the doctor, have check-ups, and discuss all concerns with the doctor | 1 | 2 | 3 | 4 | 1 | 2 | 3 | 4 |
| 10.Seek professional help when feeling sad or depressed | 1 | 2 | 3 | 4 | 1 | 2 | 3 | 4 |
| 11.Achieve and maintain a healthy body weight.  (e.g., target is waist circumference <85cm for men, <80cm for women) | 1 | 2 | 3 | 4 | 1 | 2 | 3 | 4 |
| 12.Avoid places where you might be exposed to smoke | 1 | 2 | 3 | 4 | 1 | 2 | 3 | 4 |

**Supplementary Figure 1**


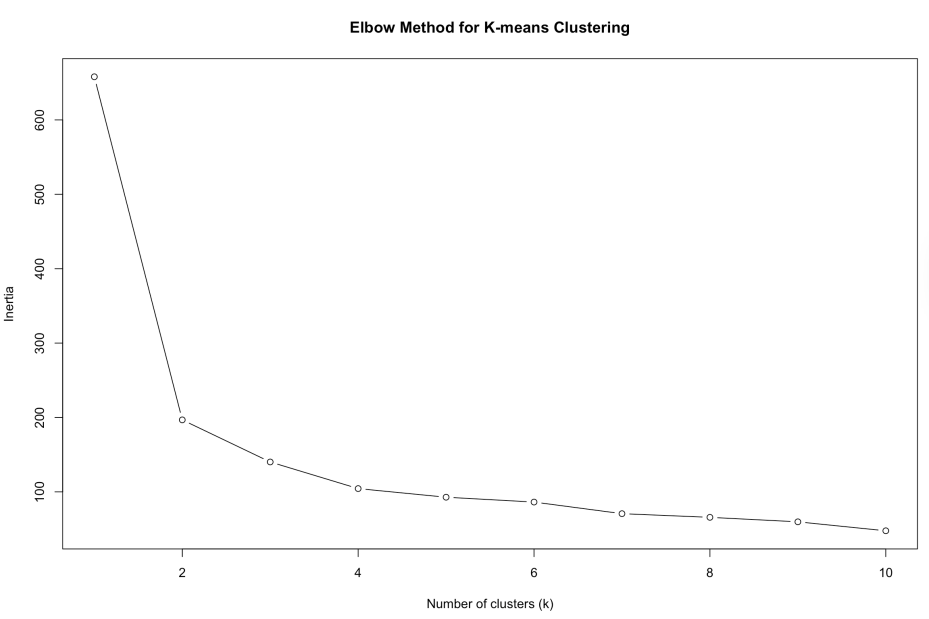


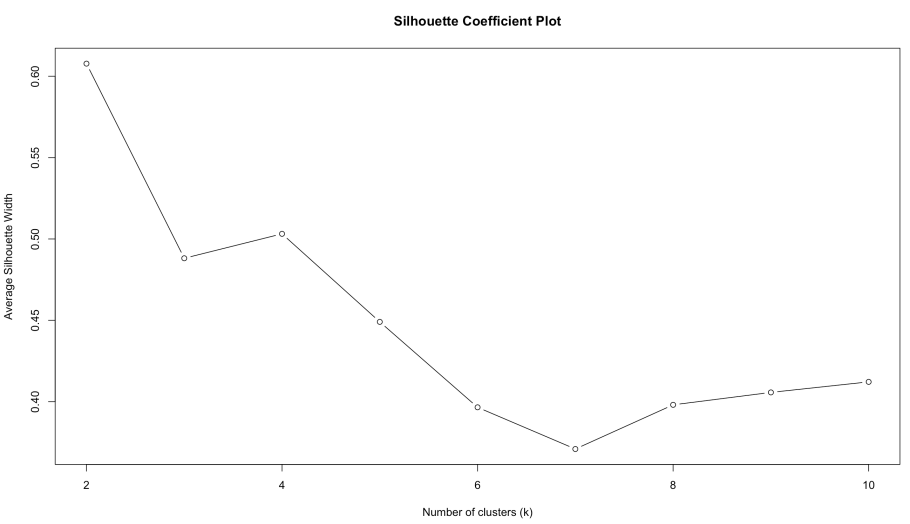


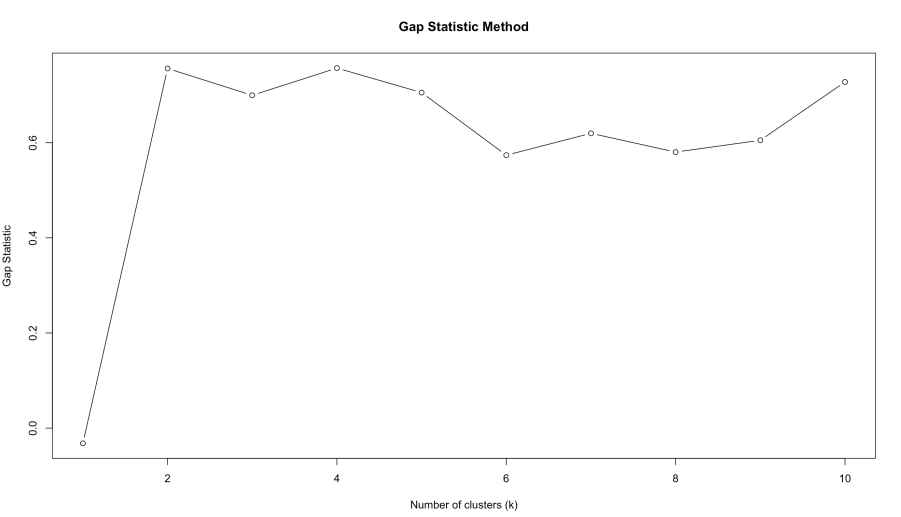

Supplement: Supplementary file 1 [file Data_Sheet_1.docx]
